# Electronic Structures and Photoelectric Properties in Cs_3_Sb_2_X_9_ (X = Cl, Br, or I) under High Pressure: A First Principles Study

**DOI:** 10.3390/nano12172982

**Published:** 2022-08-29

**Authors:** Yanwen Wu, Guangbiao Xiang, Man Zhang, Dongmei Wei, Chen Cheng, Jiancai Leng, Hong Ma

**Affiliations:** 1 Shandong Provincial Key Laboratory of Optics, Photonic Device and Collaborative Innovation Center of Light Manipulations and Applications, School of Physics and Electronics, Shandong Normal University, Jinan 250014, China; 2 Department of Physics, School of Electronic and Information Engineering, Qilu University of Technology (Shandong Academy of Science), Jinan 250353, China

**Keywords:** Cs_3_Sb_2_Br_9_, the first principles, high pressure, band structures, photoelectric properties

## Abstract

Lead-free perovskites of Cs_3_Sb_2_X_9_ (X = Cl, Br, or I) have attracted wide attention owing to their low toxicity. High pressure is an effective and reversible method to tune bandgap without changing the chemical composition. Here, the structural and photoelectric properties of Cs_3_Sb_2_X_9_ under high pressure were theoretically studied by using the density functional theory. The results showed that the ideal bandgap for Cs_3_Sb_2_X_9_ can be achieved by applying high pressure. Moreover, it was found that the change of the bandgap is caused by the shrinkage of the Sb-X long bond in the [Sb_2_X_9_]^3−^ polyhedra. Partial density of states indicated that Sb-5*s* and X-*p* orbitals contribute to the top of the valence band, while Sb-5*p* and X-*p* orbitals dominate the bottom of the conduction band. Moreover, the band structure and density of states showed significant metallicity at 38.75, 24.05 GPa for Cs_3_Sb_2_Br_9_ and Cs_3_Sb_2_I_9_, respectively. Moreover, the absorption spectra showed the absorption edge redshifted, and the absorption coefficient of the Cs_3_Sb_2_X_9_ increased under high pressure. According to our calculated results, the narrow bandgap and enhanced absorption ability under high pressure provide a new idea for the design of the photovoltaic and photoelectric devices.

## 1. Introduction

In recent years, halide perovskites have received a great amount of attention because of their excellent performances in photoelectric and photovoltaic applications [1,2,3], such as, lasers, light emitting diodes (LED), solar cell, and photocatalysis [4,5,6]. Lead halide perovskites are typical representatives due to their excellent performances. However, it is well known that lead halide perovskites suffer from low stability and high toxicity of lead in the development and applications [2,7]. Therefore, to solve the problem of toxicity, lead-free perovskites have attracted more and more attention. Since Sb^3+^ has a similar electronic structure and ionic radius as Pb^2+^, it exhibits lower toxicity and better chemical stability than the neighboring heavy metals Pb^2+^. Thus, Cs_3_Sb_2_X_9_ (X = Cl, Br, or I) becomes a superior candidate for low-toxicity polymer solar cells (PSCs) [8]. Cs_3_Sb_2_I_9_ microplates were already synthesized by the two-step chemical vapor deposition process and demonstrated superior thermal stability within a wide temperature range from 80 K to 380 K [9]. Arto Hiltunen et al. found that the average power conversion efficiency (PCE) doubled by using P3HT (poly(3-hexylthiophene-2,5-diyl)) as a hole transport material for a Cs_3_Sb_2_I_9_ solar cell with a PCE of 2.5%. The signal did not diminish during the storage for 1 month [10]. Cs_3_Sb_2_Br_9_ was first proposed as an absorber layer in PSCs to absorb solar energy by Sachchidanand et al. A maximum PCE of 15.69% for PSCs with this absorber layer was already reported [11]. The first LED with Cs_3_Sb_2_I_9_ fabricated by the vapor-anion exchange method as an emitter were reported by Anupriya Singh et al. The Cs_3_Sb_2_I_9_ film obtained by this method had exhibited wide photoluminescence spectrum with a full width at half maximum of 120 nm. Visible-infrared radiance of 0.012 W·Sr^−1^·m^−2^ at 6 V was measured in electroluminescent devices with Cs_3_Sb_2_I_9_ as the emitter layer [12]. Ummi Kalsom Noor Din et al. also synthesized lead-free, fully inorganic single crystal perovskite Cs_3_Sb_2_Br_9_ with a photoluminescence quantum yield of 23% using an inverse temperature crystallization method [13]. These excellent works have confirmed that lead-free perovskites have a promising future in the photovoltaic and photoelectronic fields.

According to the Shockley-Queisser limit, the optimal bandgap energy for photovoltaic material is 1.3–1.5 eV [14]. There are few materials that naturally conform to the ideal bandgap. The reason of low PCE for a Sb-based solar cell is that the bandgap is too large. Because of the advantage that perovskite is extremely flexible and the bandgap can be adjusted to achieve the ideal value, previous works have already reported that the absorption spectra and bandgaps (from ~1.9 to ~3.1 eV) of the A_3_B_2_X_9_ (A = Cs, MA, FA; B = As, Sb, Bi; and X = Cl, Br, I) material could be changed by atomic substitution [15]. In addition, doping is also an effective method to change the iaeal bandgap. The various concentrations of Ru^3+^ were doped into Cs_3_Bi_2_I_9_ by atomic substitution of Bi^3+^ to adjust its bandgap and optical properties. A corresponding reduction in bandgap occurs with the doping concentration of Ru^3+^. When the doping concentration is 5%, the bandgap of Cs_3_Bi_1.9_Ru_0.1_I_9_ reduced from 2.2 to 1.80 eV. The narrowing of the bandgap expands its applications in photoelectronic and photovoltaic fields [16]. High pressure can also adjust the crystal structures and physical properties without changing the chemical composition. The most important advantage of high pressure is that high pressure is reversible. Namely, when the pressure is released, the material can return to its original properties [17]. Guang Dai et al. found that the emission peak of CsPbBr_3_ perovskite quantum dots could be modulated by the applied external pressure. Detailed analysis showed that the wavelength shifting was correlated to the external pressure with a good linear relationship. They demonstrated novel perovskite quantum dots based on optical a Fabry-Perot (FP) pressure sensor for the first time [18]. Ting Geng et al. successfully narrowed the bandgap of lead-free perovskite Cs_3_Sb_2_I_9_ nanocrystals from 2.05 to 1.36 eV at 20 GPa pressure, with a measurable rate of 33.7%. The change in bandgap can be attributed to the pressure-induced compression and distortion of the lattice [19]. Experimental results obtained by Lianwei Wu et al. had reported that Cs_3_Sb_2_I_9_ can reach a bandgap of 1.32 eV at 20 GPa, successfully obtaining the ideal semiconductor bandgap (1.34 eV). As the pressure continues to increase, this material also exhibited metallic properties [17]. Therefore, high pressure can effectively adjust the structural and photoelectronic properties of perovskite materials.

Although so many theoretical and experimental works already reported, there is still a lack of systematic theoretical calculation for band structures and photoelectronic properties of Cs_3_Sb_2_X_9_ (X = Cl, Br, or I) under high pressure. In the present work, we systematically investigated the electronic structures and optical properties of Cs_3_Sb_2_X_9_ (X = Cl, Br, or I) under high pressure from 0 to 40 GPa by Density Functional Theory (DFT). Firstly, the structural properties and bandgap of Cs_3_Sb_2_X_9_ at high pressure was calculated. Then, the changes of lattice parameter and bond length under high pressure were investigated. Finally, the density of states and optical properties under high pressure were calculated. The findings in this article are expected to provide insights into the physical and chemical properties of Cs_3_Sb_2_X_9_ under high pressure and a helpful guide to develop the efficient photoelectronic and photovoltaic applications.

## 2. Computational Model and Method

The research in this paper is based on the quantum mechanical approach of DFT. DFT is often referred to as first-principles computations. The DFT calculations were performed using the projector-augmented wave (PAW) method [20], implemented in Vienna Ab-initio Simulation Package [21,22]. A single point at the generalized gradient approximation (GGA) of Perdew-Burke-Ernzerh (PBE) was used for obtaining the optimized lattice parameter, atomic position, density of state and electronic band structure [23]. On the one hand, DFT can provide microscopic explanations for experimental phenomena and identify the essential reasons of the phenomena. On the other hand, it can also predict the properties of new materials and thus provide theoretical guidance for the design of new experiments and the selection of materials for specific properties.

The layered form of Cs_3_Sb_2_X_9_ is more suitable for photovoltaic applications than the dimeric form [24]. Therefore, all calculations were chosen in layered form. The initial structure was downloaded from the Crystallography Open Database and further optimized in VASP [25,26,27]. In order to determine optical properties for Cs_3_Sb_2_X_9_, both the HSE06 hybrid exchange-correlation functional and PBE algorithms were used. We adjusted the PSTRESS in the INCAR file to change the pressure value in optimizations of the structure. The kinetic cut-off energy for the plane wave expansion was taken to be 500 eV. The structural optimizations were stopped when all the total forces on each atom were below 0.01 eV/A. The convergence criterion for the energy was set to be 10^−6^ eV [28]. The 5 × 5 × 5 k-point grid was applied to structure optimization and HSE calculated optical properties, while the dense 18 × 18 × 18 grid was applied to the PBE algorithm for calculated optical properties. To make the density of states seem smooth, 16 × 16 × 16 k-point was used for the calculations. The high-symmetry points for the band structure were taken as Γ (0 0 0), M (0.5 0 0), K (0.33 0.33 0), Γ (0 0 0), A (0 0 0.5), L (0.5 0 0.5), H (0.33 0.33 0.5) and A (0 0 0.5), respectively [24]. The crystal structures were visualized using VESTA [29].

## 3. Results and Discussion

**Figure 1:**
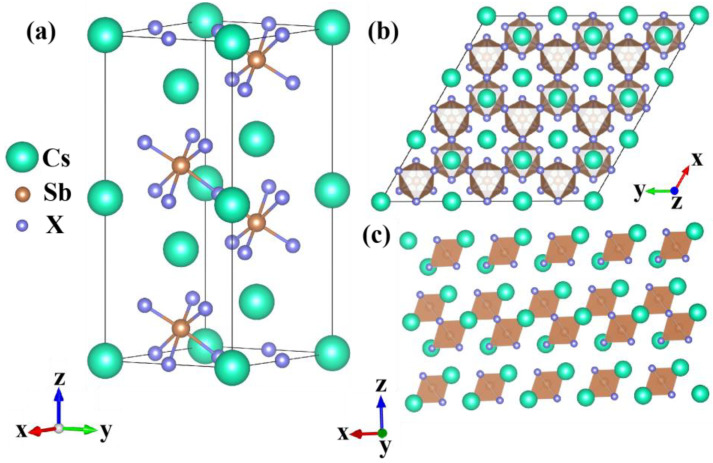
(**a**) Optimized structure of Cs_3_Sb_2_X_9_; (**b**) corresponding crystal structure of Cs_3_Sb_2_X_9_ viewed in the xy plane; (**c**) layered form Cs_3_Sb_2_X_9_.

Cs_3_Sb_2_X_9_ belongs to the trigonal crystal structure with the space group P3(—)m1 (no.164). Figure 1a, b showed the optimized crystal structure of the Cs_3_Sb_2_X_9_. Cs_3_Sb_2_X_9_ is composed of two [Sb_2_X_9_]^3−^ polyhedra in a single unit, with the two Sb^3+^ ions located on the body diagonal of the unit cell and eight top corners occupied by Cs^+^. These connected [Sb_2_X_9_]^3−^ polyhedra of the bilayer are stacked together. Cs_3_Sb_2_X_9_ is considered as layered material (Figure 1c) and exhibits triangular symmetry [28]. The optimized atomic coordinates without high pressure are listed in Supplementary Materials Table S1. In order to indicate that the structures are still stable under pressure, we calculated the pressure-dependent Gibbs free energy and show it in Supplementary Materials Figure S1.

The previous and present theoretical values of lattice parameters a, b and c bandgap energy *E_g_* for Cs_3_Sb_2_X_9_ are presented in Table 1. According to Table 1, the PBE optimized lattice parameters are in good agreement with the previously reported and experimental values. For example, the lattice parameters of Cs_3_Sb_2_Cl_9_ are 7.817, 7.817 and 9.494 Å obtained in this work, which are very close to the reported theoretical values of 7.827, 7.827 and 9.472 Å and slightly larger than those of the experimental values. As the atomic number increases from Cl to I, the lattice parameter increases in turn. In addition, the ionic radii of Cl^−^, Br^−^, and I^−^ are 1.81, 1.96 and 2.20 Å, respectively, which showed similar behavior as the lattice parameters [28]. Moreover, the bandgap energies calculated by PBE are 2.40, 2.01 and 1.55 eV without pressure for Cs_3_Sb_2_Cl_9_, Cs_3_Sb_2_Br_9_ and Cs_3_Sb_2_I_9_, respectively. Compared to the reported theoretical and experimental values, the bandgap energies calculated by PBE are small [30].

**Figure 2:**
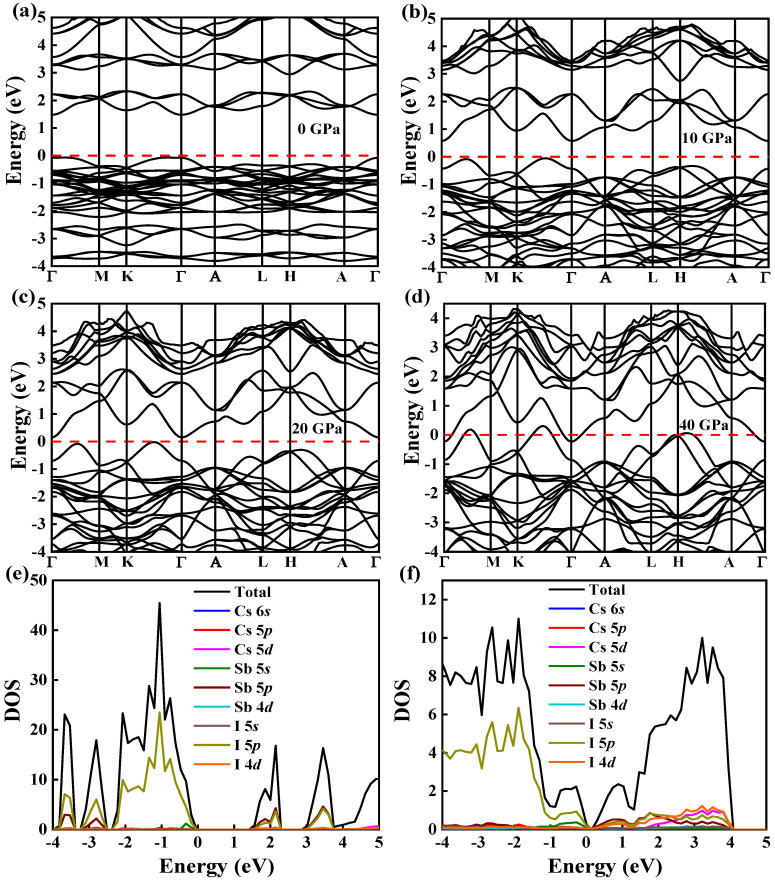
Band structures of Cs_3_Sb_2_I_9_ under different pressures of 0 (**a**); 10 (**b**); 20 (**c**); 40 GPa (**d**). The red dashed line is a guide line at 0. Total and partial density of states for Cs_3_Sb_2_I_9_ without (**e**) and with (**f**) pressure calculated by PBE functional.

The photoelectric properties of the materials are closely related with their band structures. Therefore, we calculated the band structures of Cs_3_Sb_2_X_9_ under the pressure from 0 to 40 GPa. Figure 2a–d show the band structures of Cs_3_Sb_2_I_9_ under the pressure of 0, 10, 20 and 40 GPa. As the pressure increases, the bandgap of Cs_3_Sb_2_I_9_ decreases. The bandgap under the pressure of 0, 10, 20 and 40 GPa are 1.55, 0.63, 0.17 and −0.54 eV, respectively. According to Figure 2a, Cs_3_Sb_2_I_9_ is a direct bandgap structure without pressure, with both the conduction band minimum (CBM) and the valence band maximum (VBM) at point Γ [28]. In addition, the CBM still remains at point Γ, while the VBM moves towards point K under the high pressure. The shift of the high symmetry points from Γ to the K point also appeared in the calculation of the energy band of CsYbCl_3_ at high pressure (160 GPa) [34]. As shown in Figure 2e, the valence band consists of Sb-5*s* and I-5*p*, and the conduction band mainly consists of Sb-5*p* and I-5*p* when no pressure is applied. Figure 2f shows that at a pressure of 40 GPa, the valence band still consists of Sb-5*s* and I-5*p*, while the conduction band is dominated by Sb-5*p*, I-5*p* and I-4*d*. The interaction between the I and Sb orbitals leads to a change in the high symmetry point of the energy band. The inter-conversion of direct and indirect bandgap owing to the change in composition has also been reported previously [35]. The band structures of Cs_3_Sb_2_Cl_9_ and Cs_3_Sb_2_Br_9_ under pressure are shown in Supplement Figures S2 and S3. Cs_3_Sb_2_Br_9_ is also a direct bandgap semiconductor without pressure with both CBM and VBM at point Γ, which is similar as that of Cs_3_Sb_2_I_9_. However, Cs_3_Sb_2_Cl_9_ exhibits an indirect bandgap with CBM at point A and VBM at point Γ when the pressure is not applied [24]. The CBM shifts towards point Γ, and the VBM starts moving from point Γ to point K under the pressure, still maintaining the indirect bandgap. Based on Supplement Figure S2 and S3, the bandgap energies are 2.4, 1.74, 1.28 and 0.54 eV for Cs_3_Sb_2_Cl_9_ and 2.00, 1.16, 0.70 and −0.04 eV for Cs_3_Sb_2_Br_9_ under the pressures of 0, 10, 20 and 40 GPa, respectively. The Fermi level is at 0 eV, as shown by a red horizontal dashed line. The locations of the VBM are −0.078, −0.051, −0.021 and 0.308 eV, while the position of the CBM is 1.48, 0.58, 0.15 and −0.23 eV for Cs_3_Sb_2_I_9_. The conduction band and valence band gradually move to the Fermi energy level and then overlap with each other, indicating that the material presents metallic properties [19,20]. According to Supplementary Materials Figures S2 and S3, Cs_3_Sb_2_I_9_ and Cs_3_Sb_2_Cl_9_ also exhibit significant metallicity at 24.05 and 38.75 GPa. Therefore, it is possible to obtain the desired bandgap energy by tuning the pressure.

It is noted that the bandgap calculated by PBE is smaller than that measured by the experiment. According to a previous study, the ideal bandgap of Cs_3_Sb_2_I_9_, namely 1.36 eV, was achieved under a pressure of 20 GPa [19]. Because the PBE calculation underestimates the bandgap value, the bandgap calculated in this work is only 0.17 eV at 20 GPa. According to our calculated results, Cs_3_Sb_2_X_9_ (X = Cl, Br, or I) reaches ideal bandgap values (1.34 eV) at 19.02, 6.82 and 1.14 GPa, respectively. The more theoretical values of the bandgap energies under pressure for Cs_3_Sb_2_Cl_9_, Cs_3_Sb_2_Br_9_ and Cs_3_Sb_2_I_9_ are listed in Supplementary Materials Tables S2–S4.

**Figure 3:**
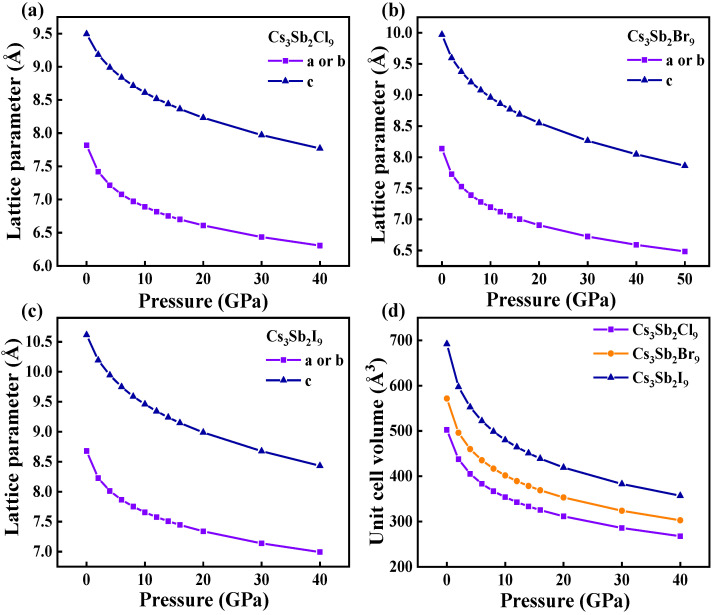
Calculated lattice parameter a, b and c of Cs_3_Sb_2_Cl_9_ (**a**); Cs_3_Sb_2_Br_9_ (**b**); Cs_3_Sb_2_I_9_ (**c**); as a function of pressure: (**d**) lattice volume of Cs_3_Sb_2_X_9_ with increasing pressure from 0 to 40 GPa.

Figure 3a–c showed the calculated lattice parameters of Cs_3_Sb_2_X_9_ under pressure. All lattice parameters displayed a decreasing trend when the pressure increased. Since Cs_3_Sb_2_X_9_ is a trigonal crystal structure, the lattice constants of the a and b are equal. Lattice constants for a and b varied in the range 7.82–6.31, 8.14–7.73 and 8.68–6.99 Å for Cs_3_Sb_2_Cl_9_, Cs_3_Sb_2_Br_9_ and Cs_3_Sb_2_I_9_, respectively. In contrast, the lattice constant in the c-direction decreases from 9.49 to 7.77 Å, 9.97 to 8.05 Å and 10.61 to 8.43 Å for Cs_3_Sb_2_Cl_9_, Cs_3_Sb_2_Br_3_ and Cs_3_Sb_2_I_3_, respectively. The decrease in lattice constant indicated that the continuous pressure induced a contraction of the unit cell. S. Idrissi et al. reported that the changes in the lattice constant cause a shrinkage in the bandgap energies [36]. Both lattice constants and bandgaps calculated in this paper for Cs_3_Sb_2_X_9_ had the same variation trend. Figure 3d showed the change in unit cell volume, which indicated that the volume of Cs_3_Sb_2_X_9_ gradually decrease. As the pressure increases, the interatomic distance decreases, leading to gradual shrinkage in both lattice constant and unit cell volume [36]. This trend follows the similar behavior as the change in lattice constants, decreasing rapidly at first and then gradually slowing down. The reason is that as the space between atoms decreases, the repulsive forces between atoms become stronger, resulting in more difficult to compress [37].

**Figure 4:**
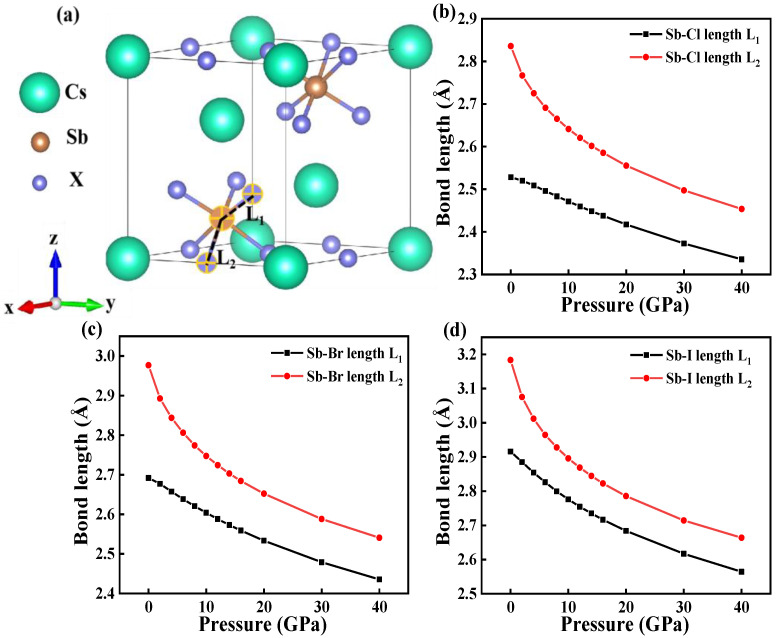
(**a**) Two types of Sb-I bonds L_1_ and L_2_. Calculated bond length as a function of pressure for Cs_3_Sb_2_Cl_9_ (**b**); Cs_3_Sb_2_Br_9_ (**c**); and Cs_3_Sb_2_I_9_ (**d**); as a function of pressure.

To explain the shrinking bandgap and lattice constant of the Cs_3_Sb_2_X_9_ under pressure, we also calculated the relation between the bond length and the pressure. As shown in Figure 4a, two different bond lengths, L_1_ and L_2_, are defined as the short and long Sb-X bonds, respectively. The bond lengths were obtained using the VESTA. Figure 4b–d presented bond length as a function of pressure for Cs_3_Sb_2_X_9_. The bond lengths of Cs_3_Sb_2_Cl_9_, Cs_3_Sb_2_Br_9_ and Cs_3_Sb_2_I_9_ increase in order without pressure, which can be explained by the increase in the atomic radius of halogen atoms [28]. Both short and long Sb-X bond lengths decrease with pressure, indicating that Cs_3_Sb_2_X_9_ are compressed under the pressure. The variation range in short bond L_1_ of the Cs_3_Sb_2_X_9_ is 2.53–2.34, 2.69–2.44 and 2.92–2.56 Å, respectively. The long bond L_2_ varies in the range of 2.84–2.45, 2.98–2.54 and 3.18–2.66 Å, respectively. The theoretically calculated bond lengths of Cs_3_Sb_2_X_9_ under the pressure from 0–40 GPa are shown in Supplementary Materials Table S5. The previous work also showed that the electronic structure of Cs_3_Sb_2_X_9_ was strongly affected by the Sb-X bond length [19,38]. In addition, L_1_ and L_2_ rapidly decrease at first and then slowly, which means that the increasing proximity of adjacent [Sb_2_X_9_]^3−^ polyhedra leads to electrostatic interactions becoming strong under high pressure [19]. As the pressure increases, the Sb and X orbitals gradually overlap, and the electronic band dispersion increases as the Sb-X bond contracts. As a result, the CBM gradually decreases towards the Fermi energy level, and the VBM starts to rise simultaneously [17]. As a result, the narrowing of the bandgap at high pressure is presented. Combined with the above analysis, the reduction in bond length leads to a shrinkage in the lattice constant and further reduces the bandgap.

**Figure 5:**
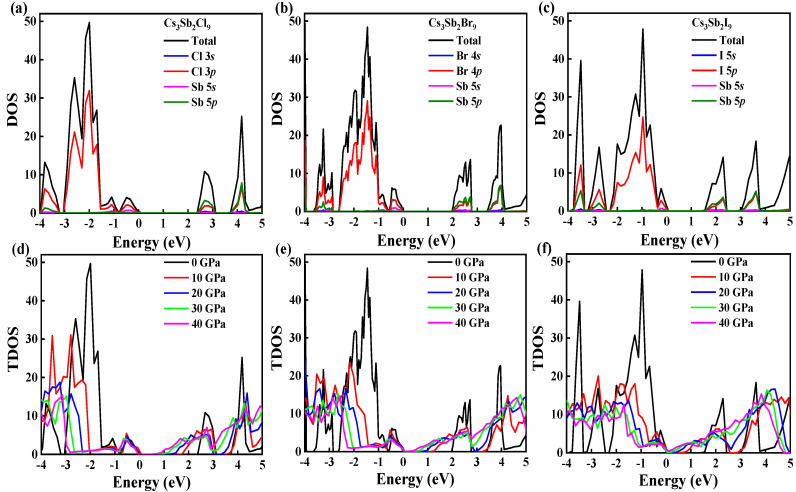
Total and partial density of states for Cs_3_Sb_2_Cl_9_ (**a**); Cs_3_Sb_2_Br_9_ (**b**); and Cs_3_Sb_2_I_9_ (**c**); are calculated by PBE functional. Total density of states of Cs_3_Sb_2_Cl_9_ (**d**); Cs_3_Sb_2_Br_9_ (**e**); and Cs_3_Sb_2_I_9_ (**f**); under different pressures.

The density of states (DOS) represents the number of electrons in the unit energy range. Much information can be obtained from a DOS diagram, such as VBM, CBM and the contribution of each orbital to the total density of states. Therefore, we calculated DOS of Cs_3_Sb_2_X_9_ with and without pressure to help us further understand the band structures. Here, the electronic configurations of 5*s*^2^5*p*^6^6*s* for Cs, 5*s*^2^5*p*^3^ for Sb, 3*s*^2^3*p*^5^ for Cl, 4*s*^2^4*p*^5^ for Br and 5*s*^2^5*p*^5^ for I are considered as valence electrons. As shown in Figure 5a–c, the partial density of states (DOS) indicates that the VBM of the Cs_3_Sb_2_X_9_ mainly originates from Sb-5*s* and X-*p*, while the CBM is dominated by both Sb-5*p* and X-*p*. DFT analysis indicates that these results are consistent with the previous reports [28]. Figure 5d-f showed the total density of states under different pressures. As the pressure increases, both VBM and CBM move towards the Fermi energy level and eventually overlap (for Cs_3_Sb_2_Br_9_ and Cs_3_Sb_2_I_9_). When the valence and conduction bands become overlapped, the material changes from semiconductor to metal, which is consistent with the previous work [39]. Additionally, according to Figure 2a–d, the conduction band dropped by 1.71 eV and the valence band rose by 0.386 eV for Cs_3_Sb_2_I_9_. Thus, the conduction band plays a major role in the narrowing of the bandgap. The electrons that make up the conduction band are mainly provided by Sb-5*p* and X-*p* and the strong anti-bonding interaction between the two electrons [17].

The absorption coefficient of perovskite is significant for photoelectric and photovoltaic applications, such as solar cells, lasers and detectors. The optical absorption coefficient of perovskite is usually expressed by the complex dielectric function, i.e., ε(ω) = ε_1_(ω) + iε_2_(ω), where ω is the frequency of the incident light and ε_1_(ω) and ε_2_(ω) are the real and imaginary parts of the complex dielectric function, respectively. Generally, ε_1_(ω) and ε_2_(ω) are used to describe the refractive and absorption behavior, respectively, which are given by the following equations [39,40]:

(1) $$\varepsilon_{1}\left(\omega \right)=1+\frac{2}{\pi }P\int_{0}^{\infty }\frac{\varepsilon_{2}\left(\omega^{\prime }\right)\omega^{\prime }d\omega^{\prime }}{\omega^{\prime }{}^{2}-\omega^{2}},\;\varepsilon_{2}\left(\omega \right)=\frac{Ve^{2}}{2\pi \hbar m^{2}\omega^{2}}\int d^{3}k{\sum_{nn\prime }\left|\langle kn|p|kn^{\prime }\rangle \right|}^{2}f\left(kn\right)*\left(1-f\left(kn^{\prime }\right)\right)\delta \left(E_{kn}-E_{kn^{\prime }}-\hbar \omega \right)$$

where *P* is the principal value of the integral, *V* represents a unit volume, e is the electron charge, *p* represents the momentum transition matrix, and *kn* and *kn*′ are the wave functions of the conduction band and valence band, respectively. The absorption coefficient, α, can be obtained by calculating the real and imaginary parts of the dielectric function [40]:

(2) $$\alpha =2\omega {\left[\frac{{\left(\varepsilon_{1}^{2}\left(\omega \right)+\varepsilon_{2}^{2}\left(\omega \right)\right)}^{1/2}-\varepsilon_{1}\left(\omega \right)}{2}\right]}^{1/2},$$

**Figure 6:**
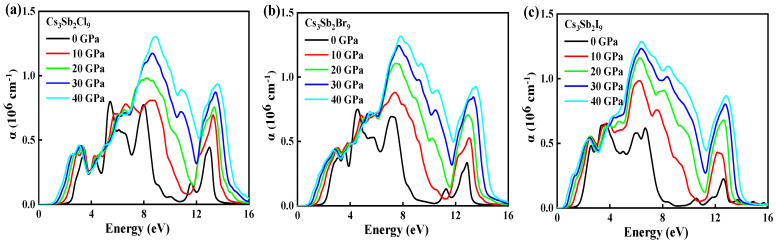
Optical spectra of Cs_3_Sb_2_Cl_9_ (**a**); Cs_3_Sb_2_Br_9_ (**b**); and Cs_3_Sb_2_I_9_ (**c**); as a function of energy are calculated by PBE functional under different pressures.

The high pressure influences the band structure, bandgap energy and the nature of the bonding of the band structure; these changes will further influence the optical absorption of Cs_3_Sb_2_X_9_. Figure 6a–c showed the absorption spectra of Cs_3_Sb_2_X_9_ as a function of photon energy at different pressures. One can see that the absorption edge shifts towards lower energies (redshift) with increasing the pressure, which is consistent with the variation of the bandgap under the pressure shown in Figure 2. The redshift of the absorption edge for CsSnCl_3_ with increasing pressure was also mentioned in a previous report by Jakiul Islam et al. [37]. Supplementary Materials Figure S3 showed the optical properties calculated using the HSE06 hybrid exchange-correlation function and employing a dense 5 × 5 × 5 k-point grid. The same variation trend is obtained for both PBE and HSE06. As the pressure increases, the gap between the conduction band and the valence band decreases or even overlaps, which facilitates the transport of carriers and thus enhances light absorption. In Figure 6a, the absorption peaks of Cs_3_Sb_2_Cl_9_ are concentrated in the ultraviolet region and can theoretically be used as an alternative material for sterilizing surgical equipment [41], whereas in Figure 6c, the absorption spectra of Cs_3_Sb_2_I_9_ fully covered the visible region and can be used in the field of solar cells [42].

The integral values of light absorption are shown in Table 2. The integral value for the absorption spectra gradually increases with the pressure. Supplement Table S6 shows the integral value of the absorption coefficient calculated using HSE06 algorithms, and the same regularity is obtained for both algorithms, which indicates that the pressure is beneficial for light absorption. As the pressure increases, the bandgap gradually decreases and approaches the ideal bandgap value, resulting in an increase in the light absorption of the material. Therefore, pressure can be used as an efficient and unpolluted method to adjust bandgap energies and enhance the absorption coefficient of Cs_3_Sb_2_X_9_ in order to develop its applications, such as solar cells, LEDs, lasers and other photovoltaic devices.

## 4. Conclusions

To summarize, we have systematically investigated the electronic structure, DOS and optical properties of lead-free Cs_3_Sb_2_X_9_ (X = Cl, Br, and I) under high pressure. Both bandgap energy and bond length of Sb-X rapidly decreased at first and then became slow with the pressure, which was led by the enhancement of the interactions of the atoms. The strong anti-bonding between Sb-5*p* and X-*p* caused a substantial downward shift of the conduction band and a slight upward shift of the valence band, resulting in a reduction or disappearance of the bandgap. The bandgap of Cs_3_Sb_2_I_9_ can be adjusted in the range of 1.55 to −0.20 eV under pressure from 0 to 40 GPa. In addition, DOS showed that the VBM of Cs_3_Sb_2_X_9_ was dominated by Sb-5*s* and Cl-3*p*, Br-4*p* and I-5*p* states, and the CBM originated from Sb-5*p* and X-*p* states. Moreover, it was found that as the pressure increases, the absorption edge shifts towards lower energy, and the light absorption coefficient increases, which is consistent with the decrease of the bandgap energy. Therefore, the high-pressure treatment can reversibly tune the bandgap and optical properties of Cs_3_Sb_2_X_9_, providing a new method for the design of the photovoltaic and photoelectronic devices.

## Figures and Tables

**Table 1:** Calculated results (Present), previous theoretical (Pre) and experimental (Exp) values of lattice parameters a, b, and c (Å), bandgap energy *E_g_* (eV) of Cs_3_Sb_2_X_9_.

Species		a	b	c	*E_g_*
Cs_3_Sb_2_Cl_9_	Present	7.817	7.817	9.494	2.40
	Pre.	7.827 [28]	7.827 [28]	9.472 [28]	2.41 [31,32]
	Exp.	7.633 [28]	7.633 [28]	9.345 [28]	3.09 [28]
Cs_3_Sb_2_Br_9_	Present	8.137	8.137	9.969	2.01
	Pre.	8.138 [28]	8.138 [28]	9.943 [28]	2.60(HSE) [28]
	Exp.	7.930 [28]	7.930 [28]	9.716 [28]	2.30 [28]
Cs_3_Sb_2_I_9_	Present	8.678	8.678	10.614	1.55
	Pre.	8.661 [33]	8.661 [33]	10.625 [33]	1.55 [33]
	Exp.	8.420 [33]	8.420 [33]	10.386 [33]	2.05 [27,33]

**Table 2:** The calculated integral value of the absorption coefficient of the Cs_3_Sb_2_X_9_ under the pressure from 0 to 40 GPa.

Pressure (GPa)	Cs_3_Sb_2_Cl_9_	Cs_3_Sb_2_Br_9_	Cs_3_Sb_2_I_9_
0	0.70 × 10^6^	1.21 × 10^6^	1.50 × 10^6^
10	0.95 × 10^6^	1.40 × 10^6^	1.85 × 10^6^
20	1.04 × 10^6^	1.44 × 10^6^	1.95 × 10^6^
30	1.10 × 10^6^	1.49 × 10^6^	2.04 × 10^6^
40	1.15 × 10^6^	1.54 × 10^6^	2.14 × 10^6^

## Data Availability

Data are contained within the article and Supplementary materials.

## Supplementary Materials

The following are available online at https://www.mdpi.com/article/10.3390/nano12172982/s1, Table S1: Optimized the atomic coordinates without high pressure. Figure S1: Calculated Gibbs free energy as a function of pressure. Figure S2: Electronic structures of Cs_3_Sb_2_Cl_9_ under different pressures of 0 (a), 10 (b), 20 (c), 40 GPa (d); Figure S3: Electronic structures of Cs_3_Sb_2_Br_9_ under different pressures of 0 (a), 10 (b), 20 (c), 40 GPa (d); Table S2: Calculated lattice parameters a, b, c, and bandgap energy *E_g_* of Cs_3_Sb_2_Cl_9_; Table S3: Calculated lattice parameters a, b, c, and bandgap energy *E_g_* of Cs_3_Sb_2_Br_9_; Table S4: Calculated lattice parameters a, b, c, and bandgap energy *E_g_* of Cs_3_Sb_2_I_9_; Table S5: Calculated bond length of Cs_3_Sb_2_X_9_; Figure S4: Optical properties are calculated by HSE06 functional for Cs_3_Sb_2_Cl_9_ (a), Cs_3_Sb_2_Br_9_ (b), and Cs_3_Sb_2_I_9_ (c); Table S6: Integrating absorption coefficient of Cs_3_Sb_2_X_9_ calculated using HSE06.
