# Supplementary material for: Electronic Structures and Photoelectric Properties in Cs3Sb2X9 (X = Cl, Br, or I) under High Pressure: A First Principles Study
Source: Nanomaterials (Basel). 2022 Aug 29;12(17):2982. doi: 10.3390/nano12172982 (PMC9457912; doi:10.3390/nano12172982)
Supplement: Supplementary file 1 [file nanomaterials-12-02982-s001.zip › nanomaterials-1846012-supplementary.pdf]

Supplementary materials for

Electronic Structures and Photoelectric Properties in $Cs_3Sb_2X_9$ (X = Cl, Br, or I) under High Pressure: A First Principles Study

Yanwen Wu [1], Guangbiao Xiang [1], Man Zhang [1], Dongmei Wei [1], Chen Cheng [1,*], Jiancai Leng [2] and Hong Ma [1,*]

[1] Shandong Provincial Key Laboratory of Optics, Photonic Device and Collaborative Innovation Center of Light Manipulations and Applications, School of Physics and Electronics, Shandong Normal University, Jinan 250014, China; yanwenwu1209@163.com (Y.W.); m17753643157@163.com (G.X.); zhangman010501@163.com (M.Z.); weidongmei@sdnu.edu.cn (D.W.)

[2] School of Electronic and Information Engineering (Department of Physics), Qilu University of Technology (Shandong Academy of Sciences), Jinan 250353, China; jiancaileng@qlu.edu.cn

* Correspondence: drccheng@sdnu.edu.cn (C.C.); mahong@sdnu.edu.cn (H.M.)

Table S1. Optimized the atomic coordinates without high pressure.

$Cs_3Sb_2Cl_9$				$Cs_3Sb_2Br_9$				$Cs_3Sb_2I_9$			
Cs_1	0.000	0.000	0.000	Cs_1	0.000	0.000	0.000	Cs_1	0.000	0.000	0.000
Cs_2	0.334	0.667	0.334	Cs_2	0.334	0.667	0.330	Cs_2	0.333	0.667	0.325
Cs_3	0.667	0.334	0.666	Cs_3	0.667	0.334	0.670	Cs_3	0.667	0.333	0.675
Sb_1	0.334	0.667	0.820	Sb_1	0.334	0.667	0.817	Sb_1	0.333	0.667	0.815
Sb_2	0.667	0.334	0.180	Sb_2	0.667	0.334	0.183	Sb_2	0.667	0.333	0.185
Cl_1	0.000	0.500	0.000	Br_1	0.000	0.500	0.000	I_1	0.000	0.500	0.000
Cl_2	0.500	0.000	0.000	Br_2	0.500	0.000	0.000	I_2	0.500	0.000	0.000
Cl_3	0.500	0.500	0.000	Br_3	0.500	0.500	0.000	I_3	0.500	0.500	0.000
Cl_4	0.178	0.357	0.670	Br_4	0.174	0.348	0.668	I_4	0.171	0.341	0.666
Cl_5	0.178	0.822	0.670	Br_5	0.174	0.826	0.668	I_5	0.171	0.829	0.666
Cl_6	0.357	0.178	0.330	Br_6	0.348	0.174	0.332	I_6	0.341	0.171	0.334
Cl_7	0.643	0.822	0.670	Br_7	0.652	0.826	0.668	I_7	0.659	0.829	0.666
Cl_8	0.822	0.178	0.330	Br_8	0.826	0.174	0.332	I_8	0.829	0.171	0.334
Cl_9	0.822	0.643	0.330	Br_9	0.826	0.652	0.332	I_9	0.829	0.659	0.334

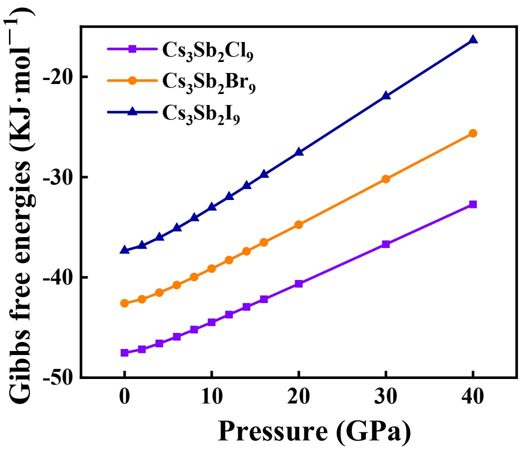

Figure S1. Calculated Gibbs free energy as a function of pressure.

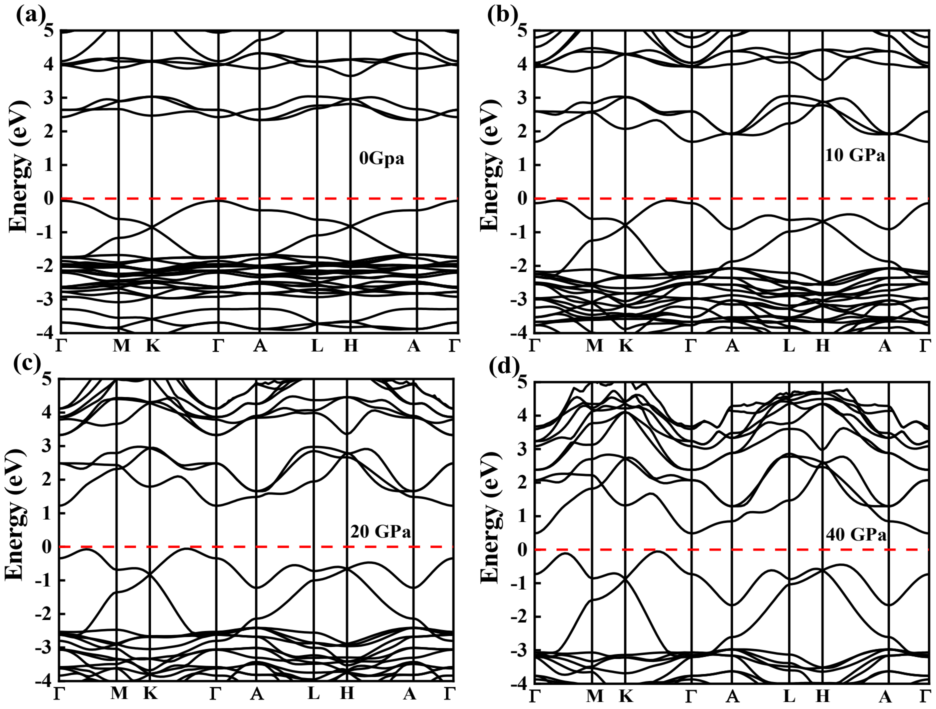

Figure S2. Electronic structures of $Cs_3Sb_2Cl_9$ under different pressure of 0 (a), 10 (b), 20 (c), 40 GPa (d).

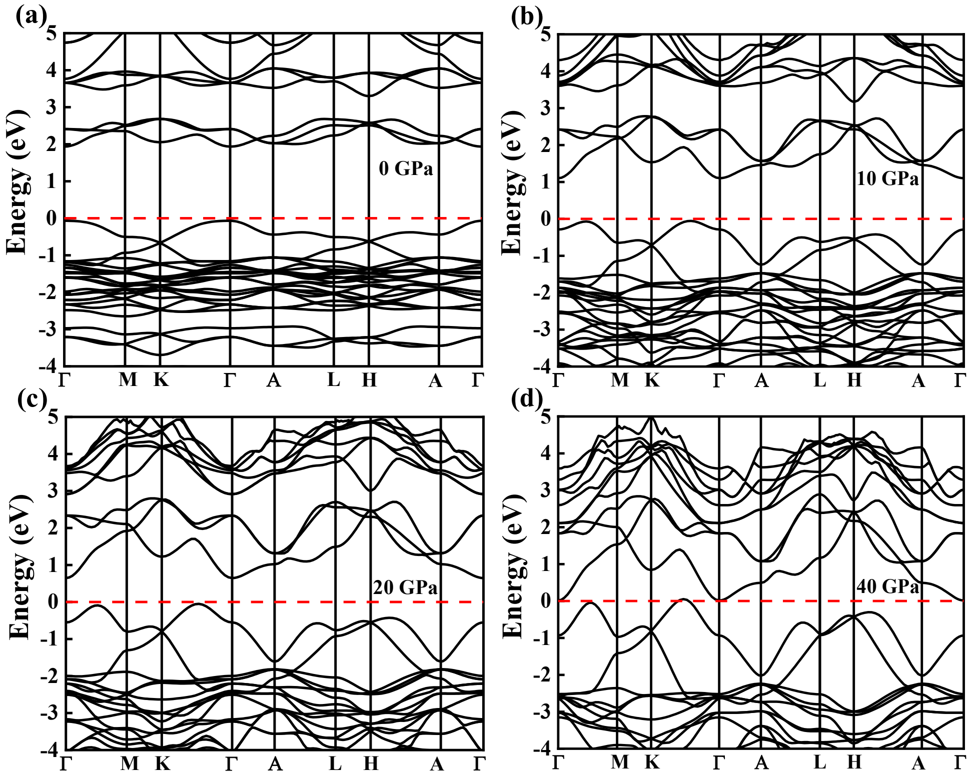

Figure S3. Electronic structures of $Cs_3Sb_2Br_9$ under different pressure of 0 (a), 10 (b), 20 (c), 40 GPa (d).

Table S2. Calculated lattice parameters a, b, c, and bandgap energy E_g of $Cs_3Sb_2Cl_9$.

Pressure (GPa)	Lattice parameter (Å)			E_g (eV)
	a	b	c	
0	7.81708	7.81708	9.49444	2.4040
2	7.41904	7.41904	9.18223	2.2445
4	7.21497	7.21497	8.98819	2.0951
6	7.07666	7.07666	8.83709	1.9648
8	6.97195	6.97195	8.71445	1.8476
10	6.88964	6.88964	8.61106	1.7414
12	6.81597	6.81597	8.51836	1.6388
14	6.75283	6.75283	8.44014	1.5458
16	6.70037	6.70037	8.36446	1.4516
20	6.60925	6.60925	8.23111	1.2811
30	6.43425	6.43425	7.97144	0.8880
40	6.30551	6.30551	7.76972	0.5397

Table S3. Calculated lattice parameter a, b, c, and bandgap energy E_g of $Cs_3Sb_2Br_9$.

Pressure (GPa)	Lattice parameter (Å)			E_g (eV)
	a	b	c	
0	8.13722	8.13722	9.96899	2.0050
2	7.72665	7.72665	9.59483	1.7273
4	7.52687	7.52687	9.37266	1.5460
6	7.38980	7.38980	9.20516	1.3978
8	7.28101	7.28101	9.07678	1.2725
10	7.19586	7.19586	8.95944	1.1583
12	7.12374	7.12374	8.85844	1.0563
14	7.05918	7.05918	8.77087	0.9589
16	7.00319	7.00319	8.68900	0.8659
20	6.90658	6.90658	8.55033	0.6973
30	6.72471	6.72471	8.26467	0.3127
40	7.72665	7.72665	8.04598	−0.0374

Table S4. Calculated lattice parameter a, b, c, and bandgap energy E_g of $Cs_3Sb_2I_9$.

Pressure (GPa)	Lattice parameter (Å)			E_g (eV)
	a	b	c	
0	8.67773	8.67773	10.61433	1.5491
2	8.22589	8.22589	10.19215	1.2147
4	8.01113	8.01113	9.94258	1.0202
6	7.86471	7.86471	9.74801	0.8689
8	7.75142	7.75142	9.58761	0.7381
10	7.65529	7.65529	9.45883	0.6250
12	7.57693	7.57693	9.34138	0.5221
14	7.50748	7.50748	9.23938	0.4263
16	7.44486	7.44486	9.14723	0.3371
20	7.33991	7.33991	8.98778	0.1701
30	7.13806	7.13806	8.67546	−0.2049
40	6.99286	6.99286	8.43188	−0.5398

Table S5. Calculated bond length under pressure of $Cs_3Sb_2X_9$.

Pressure (GPa)	$Cs_3Sb_2Cl_9$		$Cs_3Sb_2Br_9$		$Cs_3Sb_2I_9$	
	L_1 (Å)	L_2 (Å)	L_1 (Å)	L_2 (Å)	L_1 (Å)	L_2 (Å)
0	2.52822	2.83576	2.69182	2.97679	2.91589	3.18341
2	2.52011	2.76695	2.67720	2.89269	2.88518	3.07541
4	2.50878	2.72508	2.65750	2.84377	2.85423	3.01180
6	2.49600	2.69100	2.63839	2.80620	2.82609	2.96464
8	2.48341	2.66514	2.62073	2.77407	2.79969	2.92784
10	2.47136	2.64122	2.60391	2.74735	2.77611	2.89571
12	2.45934	2.62052	2.58807	2.72435	2.75454	2.86881
14	2.44847	2.60160	2.57312	2.70320	2.73520	2.84462
16	2.43768	2.58539	2.55909	2.68444	2.71650	2.82262
20	2.41731	2.55530	2.53350	2.65241	2.68390	2.78567
30	2.37273	2.49724	2.47915	2.58821	2.61684	2.71432
40	2.33547	2.45358	2.43543	2.54092	2.56433	2.66381

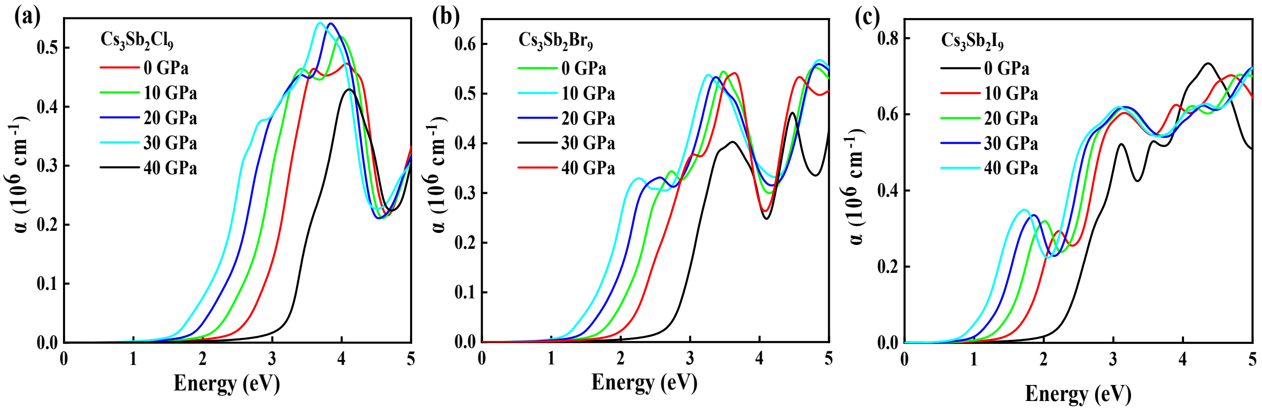

Figure S4. Optical properties under pressure calculated by HSE06 functional for $Cs_3Sb_2Cl_9$ (a), $Cs_3Sb_2Br_9$ (b), and $Cs_3Sb_2I_9$ (c).

Table S6. Integrating the absorption coefficient of $Cs_3Sb_2X_9$ calculated using HSE06.

Pressure (GPa)	$Cs_3Sb_2Cl_9$	$Cs_3Sb_2Br_9$	$Cs_3Sb_2I_9$
0	0.50×10^6	0.72×10^6	1.35×10^6
10	0.75×10^6	1.05×10^6	1.64×10^6
20	0.87×10^6	1.14×10^6	1.74×10^6
30	0.96×10^6	1.22×10^6	1.81×10^6
40	1.04×10^6	1.30×10^6	1.90×10^6
